# Metabolite Identification and Pharmacokinetic Behavior of Diaveridine in the Plasma of Pigs and Chickens Based on Radioactive Tracing Coupled With LC/MS-IT-TOF Assay

**DOI:** 10.3389/fvets.2021.799773

**Published:** 2022-01-18

**Authors:** Liye Wang, Lihua Wen, Yuanhu Pan, Zhenzhen Wang, Kaixiang Zhou, Kun Mi, Zhenli Liu, Wei Qu, Lingli Huang

**Affiliations:** ^1^National Reference Laboratory of Veterinary Drug Residues (HZAU), Huazhong Agricultural University, Wuhan, China; ^2^College of Food and Drug, Luoyang Normal University, Luoyang, China; ^3^Ministry of Agriculture (MOA) Key Laboratory for Detection of Veterinary Drug Residues, Huazhong Agricultural University, Wuhan, China; ^4^Ministry of Agriculture (MOA) Laboratory of Risk Assessment for Quality and Safety of Livestock and Poultry Products, Huazhong Agricultural University, Wuhan, China

**Keywords:** diaveridine, radioactive tracing, methodology, metabolites, pharmacokinetics

## Abstract

Diaveridine (DVD) is widely used for the prevention and treatment of coccidiosis and leucocytozoonosis infections in food-producing animals. To gain a better understanding of DVD metabolism and pharmacokinetics in healthy Landrace/Doric Cross castrated male pigs and both female and male Cobb 500 broiler chickens, a method involving radioactive tracing coupled with LC/MS-IT-TOF was developed for the identification and quantitation of DVD and its metabolites in pig and chicken plasma, and then was applied to investigate DVD pharmacokinetics. A simple MCX solid phase extraction procedure was adopted for sample preparation. After a single oral administration of ^3^H-DVD (10 mg/kg BW), three radioactive compounds (D0: DVD; D1: 3′-desmethyl-DVD; and D2: monoglucuronide of 3′-desmethyl-DVD) were identified in pig plasma, while only two radioactive compounds (D0 and D2) were identified in chicken plasma. In both species, the *C*_max_ values for all detected compounds were reached at 2 h after dosing. The *C*_max_ order was D2 (1.38 μg/ml) > D0 (0.49 μg/ml) > D1 (0.24 μg/ml) in pigs and D0 (1.55 μg/ml) > D2 (0.27 μg/ml) in chickens. The longer *t*_1/2_ (elimination half-life) of D0 contributed to the slow elimination of DVD-related compounds. The *t*_1/2β_ of D0 in pigs (66.41 h) was significantly longer than that in chickens (48.30 h), but the *t*_1/2_ of total DVD-related metabolites in pigs (42.86 h) was lower than that in chickens (56.11 h). These findings suggested that the metabolism and pharmacokinetics of DVD in pigs and chickens were significantly different, and that this would affect its effectiveness, toxicology, and food safety in these animals.

## Introduction

**Figure 1:**
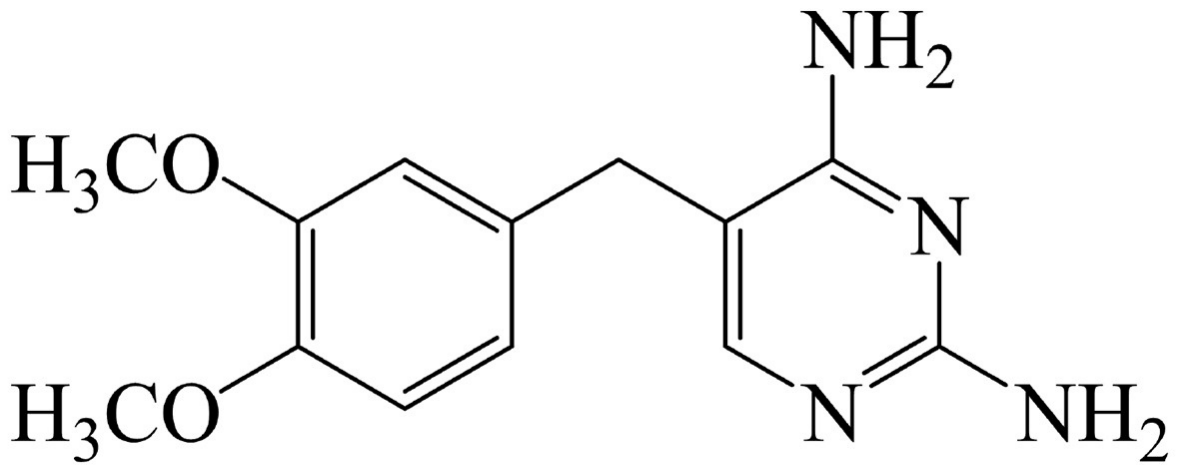
Chemical structure of diaveridine.

Diaveridine {DVD; 5-[(3′,4′-dimethoxyphenyl)methyl]-2,4-pyrimidinediamine; Figure 1} is a synthetic inhibitor of dihydrofolate reductase (DHFR), an essential enzyme in bacterial folate synthesis (1). When combined with sulfonamides or other antimicrobial agents, DVD blocks the metabolism of folic acid in bacteria by two different mechanisms thereby leading to antibacterial synergistic effects that result in a reduction in resistant strain generation (2). DVD exhibits *in vitro* antibacterial efficacy against most Gram-negative and Gram-positive bacteria, including *Escherichia coli, Clostridium* spp., *Salmonella* spp., *Staphylococcus aureus*, and *Bacillus anthracis* (3, 4). It is also noteworthy that DVD has remarkable activity against coccidia and other protozoa. Therefore, it is usually used in combination with sulfaguanidine and sulfamonomethoxine to prevent intestinal infections in the clinic (5). DVD was first considered by the Australian National Drugs and Poisons Schedule Committee in 1969 and was exempted from scheduling on the basis that it exhibited low toxicity. At the February 2003 meeting, DVD was reinstated in the Standard for the Uniform Scheduling of Drugs and Poisons (SUSDP) on the grounds that it was only used in the poultry industry (6). At present, DVD has not been licensed in the FDA and European Medicines Agency. In China, DVD has been currently permitted and extensively used for prevention and treatment of coccidiosis in poultry and rabbits and intestinal infection in livestock and poultry. For instance, sulfamethoxydiazine (SMD) and DVD premix (containing SMD and DVD 200 g and 40 g per 1000 g) was approved for prevention of intestinal bacterial infection and coccidiosis in pigs and poultry, but prohibited in laying hens. The SMD and DVD tablet (30 mg, SMD and DVD 25:5) has been approved for intestinal bacterial infection in livestock by orally administered 0.8–2 tablets at an interval of 12 h for 3–5 days. The sulfaquinoxaline and DVD premix (containing sulfaquinoxaline and DVD 200 g and 40 g per 1,000 g) was allowed to protect poultry from coccidiosis, but prohibited in laying hens (7, 8).

Toxicology studies have demonstrated that DVD is not mutagenic in either tester strain TA100 and TA98 in the presence and absence of rat S9 mix, but it was found to be mutagenic to strain TA100 after metabolic activation with hamster S9 mix (9, 10). This suggests that its metabolites may play an important role in its toxicity or activity. However, the metabolism and pharmacokinetics of DVD in food animals have only been reported in a few studies. In pig microsomes, six metabolites of DVD involving O-demethylation, α-hydroxylation, and N-oxidation were characterized using high-performance liquid chromatography combined with ion trap/time-of-flight mass spectrometry (11). In chicken, 14 metabolites of DVD involving O-demethylation, α-hydroxylation, benzene ring-hydroxylation, N-oxidation, 4′-methylation, NH_2_-glucuronidation O-demethylation-glucuronidation, and N-oxidation glucuronidation were identified in the plasma after a single oral administration of DVD by high-performance liquid chromatography linear ion trap orbitrap (LC-LTQ-Orbitrap) (12). Although mass spectrometry is well known as a method for powerful structural elucidation of drug metabolites (13), owing to the complexity of a biological sample matrix, false negative or positive results are often observed because characterization of metabolites by mass spectrometry relies only on the ion information. Metabolism studies reported for DVD indicate it can undergo extensive biotransformation in livestock, although its precise metabolism still requires further research. Our previous study identified DVD metabolites in pigs at 6 h after administration by coupling radiotracing with a LC/MS-IT-TOF method. The results showed that the method was sensitive and reliable, and a total of four radiolabeled compounds were identified from this *in vivo* experiment, of which two were new metabolites (14). Recently, a comparative pharmacokinetic study of DVD in pigs and chickens was reported following a single intravenous or oral administration (15). However, only the parent drug DVD was detected, and the data did not reveal the pharmacokinetic behaviors of any DVD-related compounds or the individual pharmacokinetic characteristics of its metabolites. It is therefore important to investigate the pharmacokinetic profiles of DVD and its metabolites to understand its disposition process and characteristics in animals.

Recently, isotopic tracing combined with online radioactivity detection and ion trap/time-of-flight mass spectrometry (MS-IT-TOF) has been shown to be a reliable analytical tool for the study of the metabolic pharmacokinetics of target compounds in an organism (16). Online radioactivity detection can be used to recognize drug-related metabolites in complex matrices and provide radiochromatographic profiles for quantitative analysis. Furthermore, MS-IT-TOF can be used to provide accurate mass spectral information for the structural elucidation of drug metabolites (17). Consequently, radiotracing combined with modern mass spectrometry technology is regarded as the gold standard for metabolic investigations by the International Cooperation on Harmonization of Technical Requirements for the Registration of Veterinary Medicinal Products (VICH) (18, 19).

In the present study, a method that included radioactive tracing coupled with LC/MS-IT-TOF was developed to identify the major metabolites of DVD in pig and chicken plasma. The method was then applied to investigate the pharmacokinetic features of DVD and its metabolites in pigs and chickens after an oral dose of ^3^H-DVD. This work should assist in revealing the pharmacokinetic parameters of DVD and its major metabolites in target animals. Moreover, these results should further enhance interpretation of the dose-effect relationship and species differences regarding DVD, in addition to providing a basis for the design of optimal doses of DVD for the treatment of livestock.

## Materials and Methods

### Chemicals

Tritium-labeled DVD (^3^H-DVD) was synthesized by the Shanghai Institute of Applied Physics, Chinese Academy of Sciences (Shanghai, China). Its chemical purity, radiochemical purity, and specific activity were ≥98%, 98.2%, and 22.08 Ci/g, respectively. The resulting solution was stably stored at −20°C for 6 months. An oral dosing formulation was prepared by accurately mixing ^3^H-DVD and non-labeled DVD at a mass ratio of 1:96 in a vehicle of 0.5% methylcellulose with a specific activity of 0.23Ci/g.

DVD reference standards (purity ≥98%, C_13_H_16_N_4_O_2_, Molecular Weight: 260.29, CAS: 5355-16-8) was supplied by Wuhan Yuancheng Science and Technology Development Co., Ltd. (Wuhan, China). Ultima Gold^TM^ was purchased from PerkinElmer Life and Analytical Sciences (Groningen, The Netherlands; Waltham, MA, USA). ^3^H liquid scintillation cocktail for online *v*.ARC radioactive detection was purchased from XenoBiotic Laboratories, Inc. (Nanjing, China). Methanol, HPLC grade was purchased from Fisher Chemicals Co., (Waltham, MA, USA). Water was purifified by a Milli-Q water purifification system (Millipore, Billerica, MA, USA). Oasis MCX (3 ml, 60 mg) cartridges were purchased from Waters Co. (Milford, MA, USA). All other chemicals and reagents were commercially obtained and of analytical grade or higher purity.

### Animals

Four healthy castrated male pigs (Landrace/Doric Cross, 60-d-old, 30 ± 2 kg) were supplied by the China Breeding Pig Testing Center of Huazhong Agricultural University (Wuhan, China). Six healthy Cobb 500 broiler chickens (three female and three male, 40-d-old, 2.5 ± 0.1 kg) were purchased from Wuhan Chia Tai chicken farm (Wuhan, China). All animals were maintained under standard environmental conditions by using normal husbandry practices and were acclimatized for 1 week. The animals were fed a commercial standard diet and water *ad libitum*, but were fasted overnight before dose administration and up to 4 h after dose administration. All animal care and experimental protocols were conducted in accordance with the Guide for the Care and Use of Laboratory Animals of Hubei Provincial Laboratory Animal Public Service Center (permit number SYXK 2013-0044) and approved by the Ethics Committee of Huazhong Agricultural University.

### Dosing and Sample Collection

Pigs (*n* = 4) and chickens (*n* = 6) were weighed individually and given a single dose of 10 mg/kg BW ^3^H-DVD with a specific activity of 0.23 Ci/g. The ^3^H-DVD solution was prepared by accurately mixing ^3^H-DVD and non-labeled DVD at a mass ratio of 1:96 and diluting it with 0.5% methylcellulose to make a homogeneous suspension with a concentration of 25 mg/ml. The suspension was administered by gastric gavage using a soft stomach tube connected to an injection syringe. About 1.5 ml of blood was collected into heparinized tubes from the anterior venae cavae of pig and the wing veins of chicken at different time points. For pigs, blood was collected before administration and 0.17, 0.33, 0.5, 0.67, 1, 1.5, 2, 4, 6, 8, 12, 24, 48, 72, 168, and 336 h after oral treatment. For chickens, blood was collected before administration and 0.25, 0.5, 1, 1.5, 2, 4, 6, 8, 12, 24, 36, 48, 72, 168, and 336 h after oral dosing. Plasma samples were collected after centrifugation at 4°C and 2,254 × *g* for 10 min and were stored at −20°C until analysis.

### Sample Preparation

Plasma samples were completely thawed at room temperature. Each sample was divided into two parts, one was used for the assay of total radioactivity, and the other was subjected to a MCX Solid Phase Extraction (SPE) procedure for the qualitative and quantitative analysis of DVD-related metabolites.

#### Sample Preparation for Phe Total Radioactivity Assay

The total radioactivity assay was conducted in accordance with a previous study with a few modifications (20). Briefly, 50 μl of plasma was mixed with 50 μl of 0.1 mol/L EDTA-Na and 50 μl of 30% H_2_O_2_ at 50°C in a shaking water bath for 2 h for bleaching. The obtained transparent liquid was mixed with 10 ml of Ultima Gold™ scintillation fluid and was analyzed directly using a Packard Tri-Carb 2900 TR liquid scintillation counter (PerkinElmer Life and Analytical Sciences, Waltham, MA, USA). The scintillation counter data were automatically corrected for counting efficiency using an instrument-stored quench curve that was drawn with a series of sealed quenched ^14^C standards. All the samples were analyzed in duplicate and the actual radioactivity of each sample was calculated by subtracting the background value from the mean radioactivity in two blank samples.

#### Sample Extraction Procedure for the DVD-Related Metabolites

A 500 μl plasma sample was adjusted to pH 2.8–3.2 with 1 ml of 1 M hydrochloric acid (HCl), and a 2 ml aliquot of the mixture was placed in a MCX cartridge preconditioned with 3 ml of methanol followed by 3 ml of 0.1 mol/L HCl water. The cartridge was washed with 3 ml of 0.1 mol/L HCl water and then eluted with 5 ml NH_4_OH/methanol (8:92, v:v). The effluent was concentrated to dryness under a stream of nitrogen at 40°C. The residue was reconstituted in 250 μl of methanol-water (1:9, v:v) before injection onto the LC-*v*.ARC/MS-IT-TOF system for metabolite identification and quantitation.

### Sample Analysis

Measurement of total radioactivity in each sample was conducted using a Packard Tri-Carb 2900 TR liquid scintillation counter. The detection time was 10 min for two cycles. The scintillation counter data were automatically corrected for counting efficiency using an instrument-stored quench curve that was drawn with a series of sealed quenched ^14^C standards. The actual radioactivity was calculated by subtracting the background value from the mean radioactivity in three blank samples.

The assay of DVD-related metabolites was conducted using a high-performance liquid chromatography system (Shimadzu Corp., Kyoto, Japan) coupled with an online isotope detector system (*v*.ARC, XenoBiotic Laboratories, Inc., Nanjing, China) and a hybrid IT/TOF-MS (Shimadzu Corp., Kyoto, Japan). The liquid chromatography system was equipped with a solvent delivery pump (LC-20AD), an auto-sampler (SIL-20AC), a DGU-20A 3 degasser, a photodiode array detector (SPD-M20A), a communication base module (CBM-20A), and a column oven (CTO-20AC). The chromatographic separation was performed using an Agilent Extend-C_18_ column [(2.1 mm × 150 mm) i.d.; 5 mm particle size; Agilent Technologies, Santa Clara, CA, USA] at 35°C, and an autosampler set at 4°C. The mobile phases consisted of solvent A (5 mM ammonium acetate containing 0.01% ammonia) and solvent B (100% methyl alcohol) delivered at a flow rate of 0.2 ml/min, with the following optimized gradient program: 0–20 min, 10–40% (B); 20–47 min, 40–90% (B); 47–49 min, 90% (B); and 49–55 min, 10% (B). The injection volume ranged from 10 to 30 μl.

Mass spectrometric detection was performed using an electrospray ionization (ESI) source operated in positive mode. Mass spectrometric analyses were carried out by scanning over the range of 100–1000 Da using data-dependent MS/MS acquisition on the suspected metabolite ions. Liquid nitrogen was used as the nebulizing gas at a flow rate of 1.5 L/min. The capillary and skimmer voltages were set at 4.5 kV and 1.6 kV, respectively. The CDL and heat block temperatures were both maintained at 200°C. MS^2^ spectra were produced using the CID of the selected precursor ions with argon as the collision gas at a relative energy of 50%. The ion accumulation time was set to 30 ms and the precursor ion isolation width was set to 1 Da. External mass calibration was carried out prior to data acquisition using direct infusion of a reference standard from 50 to 1000 Da. The reference standard consisted of 0.25 ml/L trifluoroacetic acid and 0.1 g/L sodium hydrate. The flow rate of the infusion pump was 5 ml/min. All calculated mass errors were <5 ppm after mass calibration with the reference standard. The identification of unknown metabolites was performed by comparing changes in their molecular mass, chromatographic retention time, full-scan MS/MS spectra, and accurate mass measurement with those of the parent drug. An accuracy error threshold of ± 5 mDa was set as a limit for the calculation of possible elemental compositions. Fully characterized metabolites were designated with the letter D followed by a number.

### Method Validation

The developed method was validated in terms of its ^3^H-H exchange efficiency, linearity of LSC and LC-*v*.ARC, limits of quantification (LOQ), accuracy, precision, the correlation curve between LSC and LC/LSC, column efficiency, and stability according to VICH GL 46 and GL 47.

#### ^3^H-H Exchange Efficiency

When tritium is used as a trace element, it easily undergoes an exchange with the protons in water under physiological conditions to produce tritiated water (HTO). HTO generation from ^3^H-H exchange causes the loss of radiotracer capacity in the test compound and/or its metabolites and creates ambiguous metabolism data. To assess the ^3^H-H exchange efficiency, triplicate/nonlyophilized volumetric aliquots (0.2 ml) were taken, mixed with 10 ml of scintillation fluid, and were analyzed directly by LSC. In parallel, triplicate volumetric aliquots (0.2 ml) were frozen, lyophilized in a benchtop vacuum freeze dry system (Labconco Corp., Kansas City, MO, USA), reconstituted in 0.2 ml Milli-Q H_2_O (EMD Millipore, Billerica, MA, USA), processed as previously indicated, and analyzed by LSC for total radioactivity. The observed differences in total radioactivity for each sample before and after lyophilization were attributed to the ^3^H-H exchange efficiency.

#### Recovery Rate for Sample Preparation

Recovery is an important parameter for method validation. Radioactive labeling is steadily retained in the metabolic transformation, so the extraction recovery of drug-related metabolites can easily be confirmed by comparing changes in radioactivity before and after sample preparation. In this study, the extraction recovery of total DVD-related metabolites was estimated by determining the radioactivity in the samples before and after MCX purification.

#### LSC and LC-v.ARC Linearity

Standard curves were plotted by measuring the serial volumes of ^3^H-DVD working solution (10^9^ × dilution of ^3^H-DVD standard solution) on five different days and at five concentration levels (5.13, 20.52, 82.08, 256.50, and 769.50 μg/kg) for LSC and *v*.ARC. Analyses were performed in triplicate. Correlation coefficients were used to evaluate the linearity of the calibration curve. Unknown concentrations were calculated from the calibration curve equation.

#### Limits of Detection and Limits of Quantification

Aliquots of 10 μl deionized water and 1 (262 dpm, 2.56 μg/kg), 2 (524 dpm, 5.13 μg/kg), 3 (786 dpm, 7.70 μg/kg), 4 (1048 dpm, 10.26 μg/kg), 5 (1310 dpm, 12.83 μg/kg), 6 (1572 dpm, 15.39μg/kg), 7 (1834 dpm, 17.96 μg/kg), 8 (2096 dpm, 20.52 μg/kg), 9 (2358 dpm, 23.09 μg/kg), 10 (2620 dpm, 25.65 μg/kg), and 15 μL (3930 dpm, 38.48 μg/kg) of 10^9^ × diluted ^3^H-DVD working solution were mixed with the scintillation liquid and the radioactivity was determined by LSC. Each sample was analyzed for three replicates. Limits of detection (LOD) and limits of quantification (LOQ) were calculated using triple blank activity and the lowest activity with a deviation <5% (*M*), respectively. The calculation equation was as follows:


(1) $$\begin{array}{r} LOD/LOQ=\text{M}/\left(0.2g\times 0.23Ci/g\times 2.22\times 10^{12}\right) \end{array}$$

#### Accuracy and Precision

The prepared blank samples were fortified with ^3^H-DVD at three different concentrations [5.13 μg/kg (524 dpm), 10.26 μg/kg (1048 dpm), and 20.52 μg/kg (2096 dpm)] and the radioactivity was determined by LSC. Five sets of each concentration were used in the intraday experiment. Five replicates of three concentrations were analyzed on five different days for the interday experiment. Accuracy was defined as the mean absolute recovery and was calculated by comparing the analytical results of the extracted sample with those of the standard working solution. Precision was defined by the relative standard deviation (RSD).

#### Correlation Between LSC and LC/LSC

Radioactivity in the HPLC eluates was quantified using the *v*.ARC detector. The linear correlations between cpm (*X*) from the *v*.ARC detector and dpm (*Y*) from the Tri-Carb 2900TR analyzer were determined by analyzing the radioactivity of a series of ^3^H-DVD working solutions via LSC and LC-*v*.ARC. The calibration curve was plotted by linear regression.

#### Column Efficiency

For each matrix, LSC was conducted to measure the radioactivity of the prepared sample aliquots before (*R*_1_) and after (*R*_2_) chromatographic elution. Column efficiency (*X*) was calculated as follows:


(2) $$\begin{array}{r} X=\left({\text{R}}_{2}/{\text{R}}_{1}\right)\times 100\% \end{array}$$

#### Stability

The stability of DVD and its metabolites was determined in pig plasma at 2 h after the single oral administration of ^3^H-DVD (10 mg/kg BW), and stored at −20°C for 90 d. The samples were thawed, prepared, and analyzed monthly. The measured values were compared in triplicate against freshly acquired samples.

### Data Analysis

Plasma concentration vs. time data were sequentially fitted to a non-compartmental model, using the computer program Phoenix (Version 8.2; Pharsight Corporation, Mountain View, CA, USA). The model was determined for best fit on the basis of linear trapezoidal linear interpolation. The compartment model was the best fit for both pigs and chickens. Plasma curves of DVD and its metabolites, after an oral administration of DVD, were obtained for each pig and chicken. *C*max and the time to reach *C*max (*T*max) were determined directly from the concentration vs. time curve. *t*_1/2_, *AUC*_0−_*t*__*k*__, *AUC*_0−∞_, *AUMC, MRT, C*l_B_, and *V*_d_ were calculated with the following equation:


(3) $$\begin{array}{r} t_{1/2}=\left(ln2\right)/{\text{K}}_{\text{e}} \end{array}$$

(4) $$\begin{array}{r} AUC_{0-t_{k}}=\sum_{i=1}^{k}\left(\frac{C_{i-1}+C_{i}}{2}\right)\left(t_{i}-t_{i-1}\right) \end{array}$$

(5) $$\begin{array}{r} AUC_{0-\infty }=AUC_{0-t_{k}}+\frac{C_{last}}{Ke} \end{array}$$

(6) $$\begin{array}{r} AUMC=\sum_{i=1}^{k}\frac{\left(t_{i}-t_{i-1}\right)}{2}\left(t_{i-1}\times C_{i-1}+t_{i}\times C_{i}\right) \end{array}$$

(7) $$\begin{array}{r} \text{MRT}=\text{AUMC}/\text{AUC} \end{array}$$

(8) $$\begin{array}{r} C{\text{l}}_{\text{B}}=Dose/{\text{AUC}}_{\text{inf}} \end{array}$$

(9) $$\begin{array}{r} {\text{V}}_{\text{d}}=C{\text{l}}_{\text{B}}/K_{\text{e}} \end{array}$$

Where the terminal elimination rate constant (*K*_e_) was calculated from the log-linear portion of the elimination curve using a linear regression analysis. *C*_last_ is the last observed concentration.

Differences in pharmacokinetic parameters from the two species were performed by independent sample *t*-test using IBM SPSS Statistics 25 software. *P* < 0.05 was considered as statistically significant.

## Results

### Method Validation

**Figure 2:**
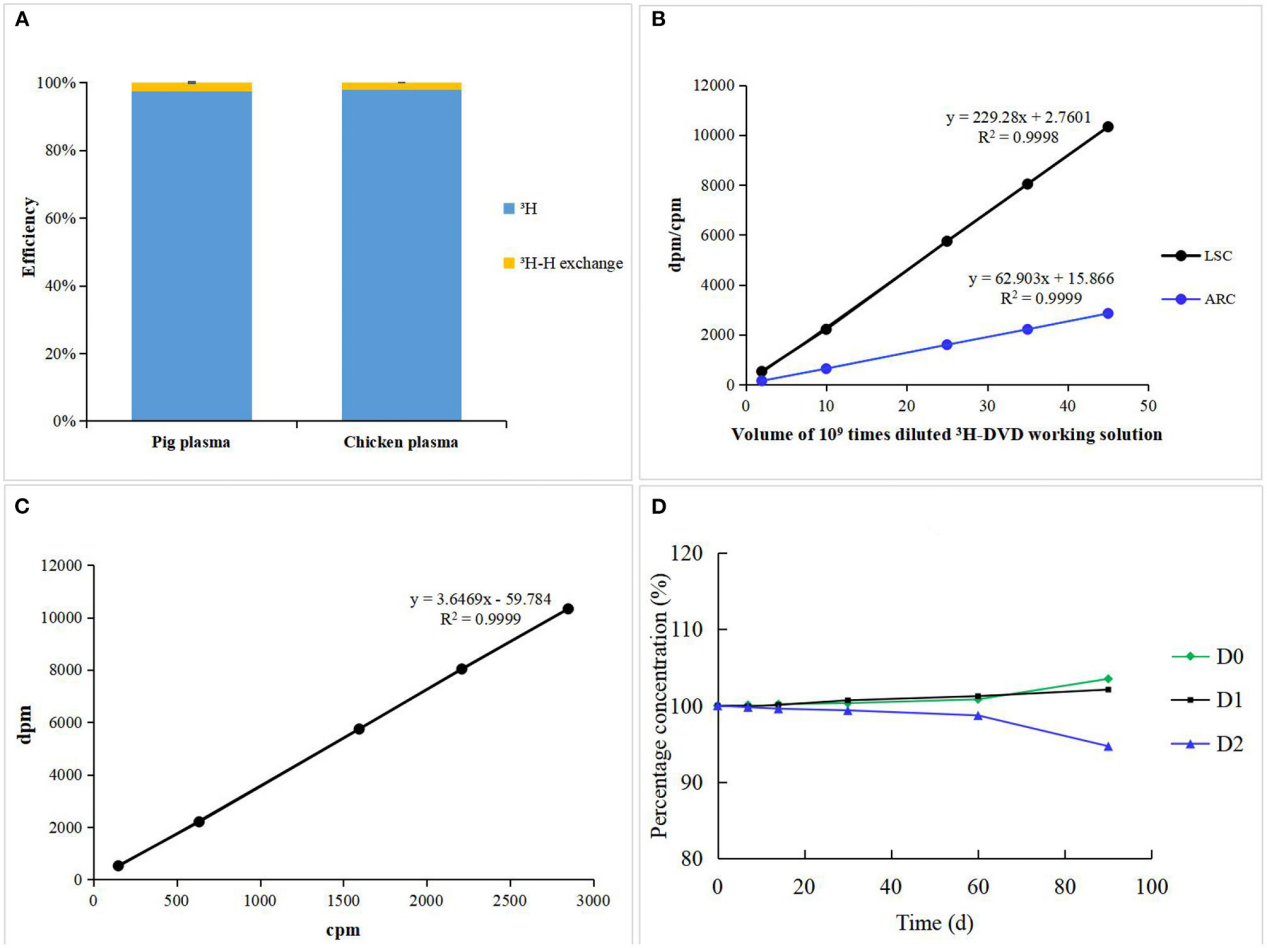
**(A)** ^3^H-H exchange efficiency of incurred pig and chicken plasma. **(B)** Standard curves and Linearity of LSC and LC-*v*.ARC. **(C)** Correlation curve between LSC and LC-*v*.ARC. **(D)** Stability of DVD and its metabolites in incurred pig plasma stored at −20°C.

To quantitatively ascertain the “^3^H-H exchange risk” of radioactive samples, the total radioactivity in pig and chicken plasma was measured before and after freeze-drying using the Tri-Carb 2900TR. No significant difference (<5%) was observed among all the samples before and after lyophilization in terms of their radioactivity suggesting that the “^3^H-H exchange risk” could be ignored (Figure 2A) (21). The extraction efficiencies of MCX sample preparation for pig and chicken plasma were 95.58 ± 3.51 and 92.18 ± 5.16%, respectively. ^3^H-DVD was serially diluted into different volumes and then was detected by LCS and LC-*v*.ARC. There was a strong linear correlation between radioactivity and response (*R*^2^ = 0.9998 and 0.9999, respectively; Figure 2B). The triple blank activity and lowest quantitative LSC limit were 270 and 524 dpm, which corresponded to a analyte concentration of 2.64 and 5.13 μg/kg, respectively. The recovery rates for the blank samples with added 1 ×, 2 ×, and 4 × LOQ radioactivity were in the range of 97.6–103.3%. The coefficient of variation was <5%. The curve (Figure 2C) delineating the correlation between LSC and LC-*v*.ARC was *y* = 3.6469*x –*59.784 (*R*^2^ = 0.9999). The liquid column recovery rate was 98.5 ± 1.7%. Hence, there was negligible chromatographic column sorption of DVD-related metabolites. The degradation rates of fresh pig plasma stored at −20 °C were <1% after 1 month, <2% after 2 months, and 2–6% after 3 months (Figure 2D). Therefore, samples must be analyzed within 2 months. The methodology and data met the requirements of VICH GL 46 and GL 47 (18, 19).

### Metabolites of DVD in Plasma of Pigs and Chickens

**Figure 3:**
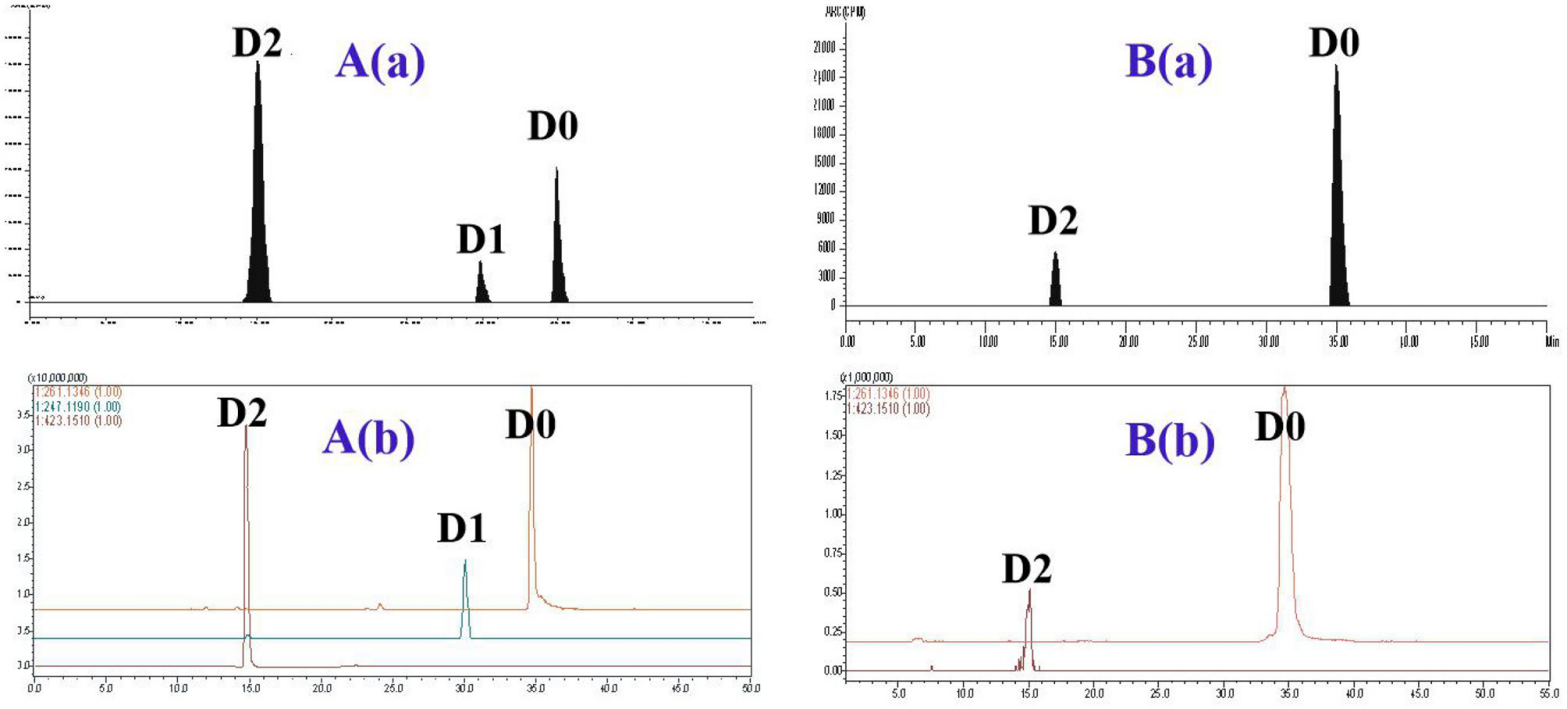
The representative HPLC radiochromatogram (RAC) and corresponding accurate extracted ion chromatogram (EIC) of pig plasma **(A)** and chicken plasma **(B)** at 2 h from a single oral administration of ^3^H-DVD at a dose of 10 mg/kg body weight: (a) RAC and (b) EIC.

**Figure 4:**
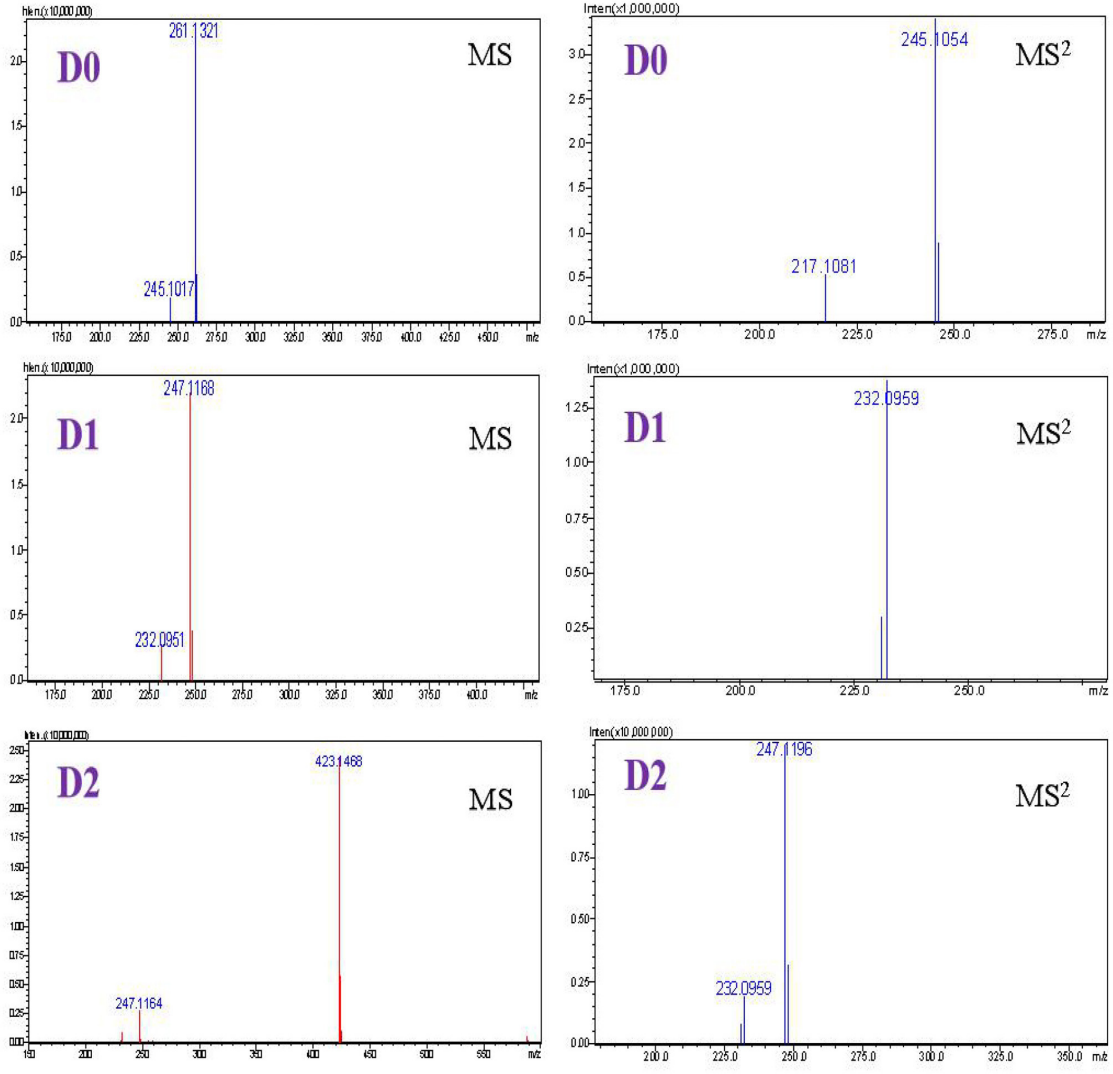
Accurate MS spectra, MS^2^ spectra of D0, D1, and D2.

The metabolite profiles of DVD in pig and chicken plasma were qualitatively identified using the LC-*v*.ARC/MS-IT-TOF system. In pig plasma, three radioactive substances were detected, named D0, D1, and D2 (Figure 3). Their chemical structures were characterized by comparing changes in their molecular mass, accurate mass measurement, and chromatographic retention time with those of the parent drug. The accurate MS/MS spectra for DVD and its metabolites are shown in Figure 4.

DVD (D0) and D2 were detected in pig plasma at 0.17 h and 0.33 h after a single oral administration (Table 1). D0, D1, and D2 were detected at 0.5 h, and were continuously observed until 48 h after administration. D2 was the predominant metabolite, followed by D0. Maximum plasma concentrations of D0, D1, and D2 were observed at 2 h after dosing at 0.49±0.02 μg/ml, 0.24±0.06 μg/ml, and 1.38±0.04 μg/ml, respectively. D2 declined rapidly over time and only D0 and D1 were observed over the period of 72–168 h; and after that, no metabolites were detected in the plasma.

**Table 1:** Concentrations (μg/ml) of DVD and its metabolites in the plasma of pigs and chickens at different time points after a single oral administration of ^3^H-DVD at a dose of 10 mg/kg body weight (*n* = 5).

**Time** **(h)**	**Pigs**				**Chickens**		
	**Total DVD-related metabolites**	**D0**	**D1**	**D2**	**Total DVD related metabolites**	**D0**	**D2**
0.17	0.11 ± 0.03^a^	0.01 ± 0.01	ND^b^	0.10 ± 0.03	/	/	/
0.25	/^c^	/	/	/	0.03 ± 0.01	0.03 ± 0.01	ND
0.33	0.34 ± 0.15	0.06 ± 0.03	ND	0.26 ± 0.11	/	/	/
0.5	0.81 ± 0.24	0.16 ± 0.05	0.12 ± 0.03	0.52 ± 0.15	0.06 ± 0.03	0.06 ± 0.02	ND
0.67	1.04 ± 0.32	0.23 ± 0.08	0.15 ± 0.02	0.61 ± 0.19	/	/	/
1	1.25 ± 0.31	0.37 ± 0.06	0.16 ± 0.04	0.69 ± 0.20	0.29 ± 0.05	0.28 ± 0.05	0.12 ± 0.03
1.5	1.96 ± 0.10	0.43 ± 0.05	0.19 ± 0.03	1.23 ± 0.04	1.15 ± 0.56	0.96 ± 0.51	0.23 ± 0.03
2	2.10 ± 0.07	0.49 ± 0.02	0.24 ± 0.06	1.38 ± 0.04	1.92 ± 0.53	1.55 ± 0.43	0.27 ± 0.09
4	1.25 ± 0.14	0.41 ± 0.04	0.16 ± 0.02	0.66 ± 0.05	1.65 ± 0.44	1.39 ± 0.38	0.18 ± 0.06
6	0.91 ± 0.10	0.31 ± 0.04	0.10 ± 0.02	0.50 ± 0.08	1.47 ± 0.48	1.22 ± 0.46	0.14 ± 0.05
8	0.79 ± 0.07	0.28 ± 0.04	0.08 ± 0.01	0.41 ± 0.07	1.04 ± 0.30	0.94 ± 0.28	0.09 ± 0.03
12	0.71 ± 0.09	0.26 ± 0.04	0.08 ± 0.01	0.35 ± 0.06	0.89 ± 0.23	0.84 ± 0.24	0.04 ± 0.01
24	0.61 ± 0.13	0.24 ± 0.04	0.06 ± 0.01	0.27 ± 0.08	0.72 ± 0.23	0.69 ± 0.22	ND
36	/	/	/	/	0.40 ± 0.05	0.39 ± 0.05	ND
48	0.33 ± 0.02	0.21 ± 0.02	0.02 ± 0.01	0.09 ± 0.01	0.28 ± 0.04	0.32 ± 0.09	ND
72	0.18 ± 0.02	0.17 ± 0.02	ND	ND	0.16 ± 0.01	0.15 ± 0.02	ND
168	0.06 ± 0.02	0.06 ± 0.02	ND	ND	0.09 ± 0.03	0.09 ± 0.03	ND
336	ND	ND	ND	ND	ND	ND	ND

a *Mean ± standard deviation*.

b *The concentration of compound was below the LOQ*.

c *Not detected*.

***D0***. D0 showed a protonated molecular ion signal at m/z 261 (Supplementary Figure 1). The MS^2^ spectrum of D0 generated fragment ions at m/z 245, which were formed by the loss of a methyl radical from m/z 261. The fragment ion at m/z 217 resulted from the loss of 28 Da at m/z 245 and corresponded to a CO loss. The formation of m/z 123 occurred by cleavage of a methylene carbon from the m/z 217 species. These results were compared with those of the reference substance. D0 was inferred to be DVD.

***D1***. D1 had a protonated molecule ion signal at m/z 247, which was 14 Da lower than that of D0, suggesting that D1 is an O-demethylation metabolite of DVD (Supplementary Figure 2). Protonated D1 fragmented to yield an abundant product ion at m/z 232 through the loss of a methyl group from the parent ion. Considering the DVD structure, methane loss may have occurred at the C-3′ or C-4′ methoxy groups. This pattern was similar to that of the analog trimethoprim (TMP) (22). The 3′-demethylated TMP content (>30%) was considerably higher than the 4′-demethylated TMP content (1%) in urine at 8 h after a single oral administration dose of 10 mg/kg BW TMP. A three-dimensional structure simulation analysis showed that the stability of the 4′-hydroxymethyl group was higher than that of the 3′-hydroxymethyl group. Therefore, we assumed that D1 most likely corresponded to 3′-OH-DVD.

***D2***. Metabolite D2 exhibited a protonated molecular ion signal at m/z 423, which was 176 Da higher than that of D1, implying that D2 was a glucuronide metabolite of D1 (Supplementary Figure 3). However, the position of glucuronidation could not be defined owing to limited mass spectrum information. Compared with the DVD structural analog brodimoprim, the compound resulting from glucuronidation of demethyl-brodimoprim was identified as demethyl-brodimoprim-O-glucuronide owing to the fact that the hydroxyl in the benzene ring was more reactive toward glucuronidation than the amino groups in the pyrimidine ring (23). We proposed that demethylation took place first, followed by formation of a hydroxyl, which was then believed to conjugate with glucuronic acid. Therefore, D2 was tentatively identified as demethyl-diaveridine-O-glucuronide.

In chicken plasma, two radioactive substances were detected and identified to be D0 and D2 (Figure 3). Only D0 was detected in the plasma 0.25 h and 0.5 h after the dose administration. From 1 to 12 h after dose administration, D0 and D2 were detected while only D0 was observed from 24 to 168 h. After that, the concentration of D0 was lower than the quantifiable level. Overall, D0 was the primary radioactive component. Maximum plasma concentrations of D0 and D2 were observed at 2 h after dosing at 1.55 ± 0.43 and 0.27 ± 0.09 μg/ml, respectively (Table 1).

### Pharmacokinetics of DVD and Its Metabolites in Pig and Chicken Plasma

**Figure 5:**
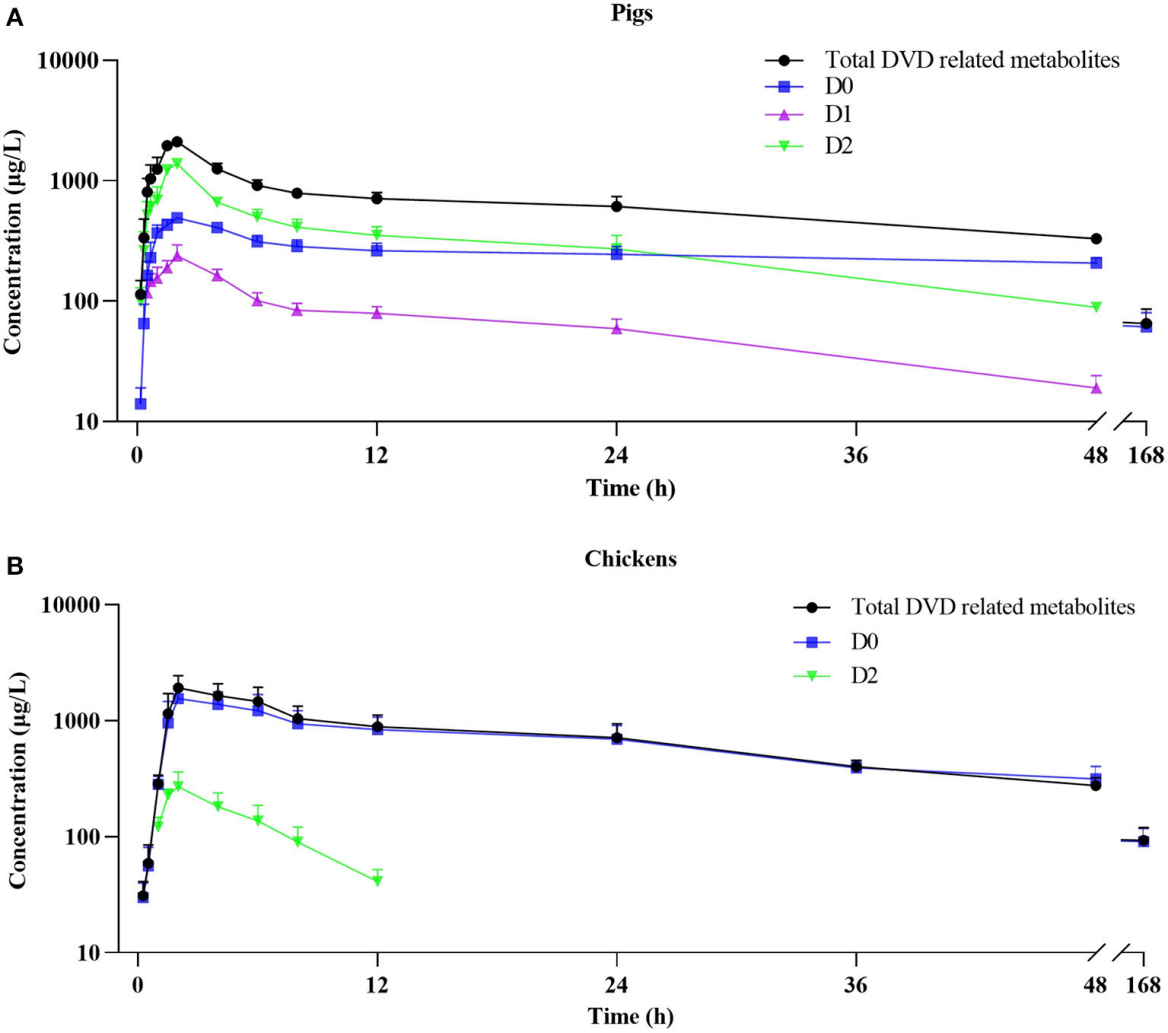
Plasma concentration-time curve of DVD related-metabolites after a single oral administration of ^3^H-DVD at a dose of 10 mg/kg body weight: **(A)** pigs and **(B)** chickens.

The mean plasma concentration–time curves for DVD and its major metabolites in pig and chicken plasma after an oral dose are shown in Figure 5. Table 2 displays the primary pharmacokinetic parameters obtained using non-compartment model analysis. After a single oral dosing in pig, the concentrations of DVD and its three related metabolites D0, D1, and D2 reached a peak (C_max_) at 2 h, at levels 2.10, 0.49, 0.24, and 1.38 μg/ml, respectively. Although the concentration of D2 was significantly higher than that of D0, D2 was rapidly eliminated. The t_1/2_ values of total DVD and its three related metabolites were 42.86, 66.41, 17.86, and 18.26 h, respectively. The AUC_0−__∞_ of total DVD and its three related metabolites were 53.77, 33.94, 3.72, and 17.58 μg·h/L, respectively; the MRT values were 54.98, 92.96, 22.93, and 23.01 h, respectively.

**Table 2:** Pharmacokinetic parameters of DVD and its metabolites in the plasma of pigs and chickens after oral administration of ^3^H-DVD at a dose of 10 mg/kg body weight.

**Parameters**	**Pig**				**Chicken**		
	**Total DVD related metabolites**	**D0**	**D1**	**D2**	**Total DVD related metabolites**	**D0**	**D2**
t_1/2_ (h)	42.86 ± 1.99	66.41 ± 10.06	17.86 ± 1.30	18.26 ± 1.70	56.11 ± 16.74	48.3 ± 12.82	3.56 ± 0.44
T_max_ (h)	2.0 ± 0.0	2.0 ± 0.0	2.0 ± 0.0	2.0 ± 0.0	2.0 ± 0.0	2.0 ± 0.0	2.0 ± 0.0
C_max_ (μg/ml)	2.10 ± 0.06	0.49 ± 0.02	0.24 ± 0.05	1.38 ± 0.04	1.92 ± 0.48	1.55 ± 0.39	0.27 ± 0.08
AUC_0−∞_ (μg·h/ml)	53.77 ± 7.06	33.94 ± 5.81	3.72 ± 0.62	17.58 ± 2.32	58.76 ± 10.15	55.29 ± 11.52	1.73 ± 0.44
V_d_ (L/kg)	11.63 ± 1.01	28.34 ± 0.85	70.38 ± 6.67	15.39 ± 3.28	14.78 ± 6.36	13.54 ± 5.94	32.45 ± 12.36
Cl_B_ (L/hr/kg)	0.19 ± 0.02	0.30 ± 0.05	2.76 ± 0.46	0.58 ± 0.08	0.18 ± 0.03	0.19 ± 0.04	6.15 ± 1.58
MRT_Last_ (h)	41.15 ± 1.23	57.27 ± 1.72	15.23 ± 0.25	14.91 ± 0.41	42.37 ± 1.43	43.74 ± 2.42	4.66 ± 0.17
MRT_0−∞_ (h)	54.98 ± 4.11	92.96 ± 12.63	22.93 ± 1.28	23.01 ± 1.48	68.21 ± 9.50	66.16 ± 10.74	6.20 ± 0.18

*t_1/2_, elimination half-life; T_max_, time to C_max_ from time zero; C_max_, maximum drug concentration; AUC_0−∞_, the area under the plasma concentration–time curve from 0 to ∞; V_d_, apparent volume of distribution; Cl_B_, total body clearance; MRT, mean residence time*.

Following a single oral dosing, D0 and D2 were detected in chicken plasma. Similar to those of the pig, the C_max_ of DVD and its related metabolites D0 and D2 were observed at 2 h after the administration with average concentrations of 1.92±0.53, 1.55±0.43, and 0.27±0.09 μg/ml, respectively. Compared with the quick elimination of D2 (t_1/2_ = 3.56 h), the elimination of D0 from the plasma occurred slowly with a t_1/2_ of 48.30 h. The AUC_0−∞_ of total DVD and its related metabolites D0 and D2 were 58.76, 55.29, and 1.73 μg·h/L, respectively; and the MRT values were 68.21, 66.16, and 6.20 h, respectively.

Statistical analysis was used to evaluate differences in pharmacokinetics parameters between the total DVD related-metabolite and the same metabolites in the plasma of the two species. Results showed that the MRT_0−∞_ value of total DVD-related metabolites in chickens was markedly longer than that in pigs (P < 0.05), while other observed pharmacokinetic parameters were without notable differences between the two species. For the metabolites of D0 and D2, there were no significant difference for t_1/2_ of D0 between the two species. However, there were significant differences for other observed pharmacokinetic parameters. These results suggested that DVD underwent extensive metabolism and presented different pharmacokinetic characteristics in pigs and chickens.

## Discussion

Pharmacokinetic studies are critical steps for understanding dynamic characteristics and optimizing dosage regimens for systemically acting antimicrobial drugs (24). Because drug metabolism is often unknown, general pharmacokinetic studies usually focus on the drug prototype and ignore the pharmacokinetic behavior of drug-related metabolites that may have some pharmacological or toxicological activity (25). Landrace/Doric Cross castrated male pigs and Cobb 500 broiler chickens, as the main commercial breeds used in animal husbandry in China, were selected in this study. Metabolites of DVD in pig and chicken plasma were identified and their pharmacokinetic behaviors were studied by a method that included radioactive tracing coupled with LC/MS-IT-TOF. Because of the traceable characteristics of the radioactive label, DVD-related metabolites can be identified and quantified by the LC-v.ARC system, and LC/MS-IT-TOF can provide accurate mass spectra information for the metabolic illustration (17). Oasis MCX, a strong cation-exchange cartridge, is preferred for the extraction of basic analytes in complex matrices, and is the most commonly employed sorbent for the clean-up of DVD structural analogs such as TMP, DVD, and ADP (26–28). In the present study, a routine MCX purification procedure was performed for plasma samples, and extraction efficiency was evaluated by comparing total radioactivity before and after sample preparation. The results suggested that the extraction recoveries of DVD-related metabolites in pig and chicken plasma were more than 90%.

Metabolism plays an important role in the kinetic elimination of DVD in pigs and chickens. In pig plasma, in addition to unmodified DVD, two radioactive metabolites, 3-desmethyl-DVD (D1), and the monoglucuronide of 3-desmethyl-DVD (D2) were detected. The metabolism of brodimoprim (BMP), an analog of DVD, has been studied in rats using NMR and mass spectrometry. Unchanged BMP, methylated BMP, and the glucuronide of BMP were found in plasma after a single oral dose of 300 mg/kg BW (23). Similarly, the metabolism of ^14^C-trimethoprim (^14^C-TMP) has been investigated in pigs of various ages after i.v. administration. TMP, demethylated TMP, and the glucuronide of TMP were detected in the plasma; D0 was the most prominent component in 1-day-old pig plasma, while the TMP glucuronide conjugate was the major component in 8-day-old pig plasma (29). These results were consistent with the findings in the current study. In the in vitro metabolism study of DVD using pig liver microsomes, D1 was detected, but no D2 was found (11). This suggests that the liver is a vital organ for the demethylation of DVD, and glucuronide conjugation may occur in the extrahepatic metabolic system. In chicken plasma, only D0 and D1 were detectable; D2 was not observed. Differences in the metabolites between pigs and chickens were probably caused by different species-related expression in the enzymes responsible for the biotransformation of DVD (30); this requires further study.

The disposition curves were described by a noncompartmental model based on SMT. The detailed pharmacokinetic parameters of DVD and its metabolites in pigs and chickens were investigated in this study using an LC-*v*.ARC/MS-IT-TOF-based method. Following oral administration, DVD was well absorbed and reached *C*_max_ at 2 h with average concentrations of 0.49 and 1.55 μg/ml in pig and chicken plasma, respectively. Using an HPLC-UV method, the *C*_max_ values for DVD were similar (0.43 and 1.45 μg/ml in pigs and chickens, respectively, in previous studies), but the *T*_max_ values were slightly different (1.04 and 3.25 h in pigs and chickens in previous studies). Moreover, in the present study, the *t*_1/2_ values for DVD in the two species were significantly longer than those of a previous study (15). These distinctions were probably related to the different sample collection time points as well as the different types of analytical methods used in the two investigations. Detection ability of the high-performance liquid chromatography–ultraviolet (HPLC-UV) method is limited. For example, DVD was detected at 168 h in pig and chicken plasma in our study, whereas it was only detected at 6 h in pigs and at 24 h in chickens using a HPLC-UV by Li et al. (15). Although radioactive tracing has sufficient sensitivity for metabolite discovery and quantification, the accuracy of quantification is questionable owing to the absence of reference standards. Slight errors in quantitative concentrations will lead to significant differences in pharmacokinetic parameters. Thus, it is necessary to prepare metabolite references of DVD for further investigation.

Notably in pigs, D1 was not modified from its basic 2,4-diaminopyrimidine structure and would therefore be expected to have some antibacterial activity (31). Consequently, it may be necessary to consider the metabolite D1 for calculation of the appropriate dosage of DVD. The identification of D0 combined with the potentially active metabolite D1 in plasma suggested that although DVD is usually used as an intestinal infection drug (32), it will have a better therapeutic effect as a systemic drug. The elimination half-life differed significantly among the detected compounds; this was characterized by the rapid elimination of metabolites D1 and D2 and the slow elimination of D0. The differences may be attributed to the biotransformation, which could increase the hydrophilicity of the parent drug, facilitating its removal from organs and tissues (33). Our results showed that the *t*_1/2_ of DVD in chicken was slightly longer than that in pig after oral dosing. This finding is consistent with those found for TMP (34). The slow elimination of DVD in chicken plasma indicates a longer residue in tissues and organs. Li and Bu (3) confirmed that withdrawal period of DVD in muscle, liver, kidney and skin with fat were 4.77, 4.94, 6.74. and 4.58 d, respectively (3). Compared with muscle and skin with fat, the kidney has a longer elimination half-life of DVD, followed by the liver. This could be explained by the kidneys and liver having a greater blood supply than muscle and skin with fat (35).

Similar to aditoprim (ADP), the types and concentrations of metabolites in pigs were significantly higher than those in chickens, indicating that the metabolic activity in pigs regarding 2,4-diaminopyrimidines was stronger than that of chickens. The concentration of total DVD related metabolites in pig plasma was significantly higher than that in chickens, which may be attributed to the weak absorption of DVD by the short intestinal structure of chickens. However, due to the weak metabolic ability of DVD in chickens, the concentration of parent D0 in plasma was significantly higher than that found in pigs. This observation could be associated with the long elimination half-time of DVD in chickens, which is caused by the stronger lipotropy of the parent drug. These studies demonstrate that there are significant interspecies differences in the metabolic and kinetic characteristics of DVD in pigs and chickens, that will affect the pharmacological, toxicological, and residue behavior of DVD in the two species (36).

## Conclusion

In the current work, reliable radioactive tracing coupled with a LC/MS-IT-TOF method was developed for the identification and determination of DVD and its metabolites in pig and chicken plasma and was successfully applied to a pharmacokinetic study where ^3^H-DVD was administered as a single oral dose. DVD was rapidly metabolized in both pigs and chickens, and three DVD-related (D0, D1, D2) and two DVD-related metabolites (D0, D2) were detected in the plasma of pigs and chickens, respectively. The metabolic capacity of pigs toward DVD was stronger than that of chickens. D0 contributes to the long half-life of the total DVD-related compounds in the two species. The plasma results revealed the dynamic process of DVD metabolism in pig and chicken and should help to improve the theoretical basis for dosage optimization of DVD in these animals. Evaluation of the complete metabolism, distribution, and depletion of DVD in different species needs to be further investigated in the future.

## Data Availability Statement

The original contributions presented in the study are included in the article/Supplementary Material, further inquiries can be directed to the corresponding authors.

## Ethics Statement

The animal study was reviewed and approved by all animal care and experimental protocols were conducted in accordance with the Guide for the Care and Use of Laboratory Animals of Hubei Provincial Laboratory Animal Public Service Center (Permit No. SYXK 2013-0044) and approved by the Ethics Committee of Huazhong Agricultural University.

## Author Contributions

LWa contributed manuscript preparation. ZW revised the manuscript. KZ and KM analyzed the data. LWe and ZL performed all the experiments. WQ revised the manuscript and interpreted the statistical analysis. LH and YP contributed to the conception and designed the studies. All authors listed have made a substantial, direct, and intellectual contribution to the work and approved it for publication.

## Funding

This work was partly financially supported by the Coordinated Research Project of International Atomic Energy Agency (2257/D52043) and the Key Scientific and Technological Project of Henan Province Department of China (No. 202102110103).

## Conflict of Interest

The authors declare that the research was conducted in the absence of any commercial or financial relationships that could be construed as a potential conflict of interest.

## Publisher's Note

All claims expressed in this article are solely those of the authors and do not necessarily represent those of their affiliated organizations, or those of the publisher, the editors and the reviewers. Any product that may be evaluated in this article, or claim that may be made by its manufacturer, is not guaranteed or endorsed by the publisher.

## Abbreviations

^3^H-DVD: tritium-labeled diaveridine
AUC_0−∞_: the area under the plasma concentration–time curve from 0 to ∞
BW: body weigh
CID: collision-induced dissociation
CDL: curved desorption line
Cl_B_: total body clearance
*C*_max_: maximum drug concentration
D1: 3′-desmethyl-DVD
D2: monoglucuronide of 3′-desmethyl-DVD
DVD/D0: diaveridine
HPLC: high-performance liquid chromatography
LC/MS-IT-TOF: liquid chromatography combined with hybrid ion trap/time-of-flight mass spectrometry
LC-LTQ-Orbitrap: high-performance liquid chromatography/linear ion trapped orbitrap
LSC: liquid scintillation counter
MRT: mean residence time
SPE: solid phase extraction
*t*_1/2_: elimination half-life
*T*_max_: time to *C*_max_ from time zero
*v*.ARC: online isotope detector system
*V*_d_: apparent volume of distribution
VICH: International Cooperation on Harmonization of Technical Requirements for the Registration of Veterinary Medicinal Products.
