# Supplementary material for: Metabolite Identification and Pharmacokinetic Behavior of Diaveridine in the Plasma of Pigs and Chickens Based on Radioactive Tracing Coupled With LC/MS-IT-TOF Assay
Source: Front Vet Sci. 2022 Jan 18;8:799773. doi: 10.3389/fvets.2021.799773 (PMC8803906; doi:10.3389/fvets.2021.799773)
Supplement: Supplementary file 1 [file Data_Sheet_1.doc]

**Metabolite Identification and Pharmacokinetic Behavior of Diaveridine in the Plasma of Pigs and Chickens Based on Radioactive Tracing Coupled with LC/MS-IT-TOF Assay**

[[1]](#footnote-2)

**Liye Wang^1,2^, Lihua Wen^1^, Yuanhu Pan^1,3,4^, Zhenzhen Wang^2^, Kaixiang Zhou^1,3,4^, Kun Mi^1,3,4^, Zhenli Liu^1,3,4^, Wei Qu^1,3,4*^ and Lingli Huang^1,3,4*^**

^1^National Reference Laboratory of Veterinary Drug Residues (HZAU), Huazhong Agricultural University, Wuhan, Hubei 430070, PR China

^2^College of Food and Drug, Luoyang Normal University, Luoyang, Henan 471934, PR China

^3^Ministry of Agriculture (MOA) Key Laboratory for Detection of Veterinary Drug Residues, Huazhong Agricultural University, Wuhan, Hubei 430070, PR China

^4^Ministry of Agriculture (MOA) Laboratory of Risk Assessment for Quality and Safety of Livestock and Poultry Products, Huazhong Agricultural University, Wuhan, Hubei 430070, PR China

* **Correspondence:**

Wei Qu

qw@mail.hzau.edu.cn

Lingli Huang

[huanglingli@mail.hzau.edu.cn](mailto:huanglingli@mail.hzau.edu.cn)

**FIGURE S1** Accurate MS, MS^2^ spectra, proposed fragmentation pathways of D0 (diaveridine)

**FIGURE S2** Accurate MS spectra, MS^2^ spectra, and proposed fragmentation pathway of D1 (3′-demethyl-diaveridine)

**FIGURE S3** Accurate MS spectra, MS^2^ spectra, and proposed fragmentation pathway of D2 (3′-demethyl-diaveridine-O-glucuronide)

1. **Abbreviations**: ^3^H-DVD, tritium-labeled diaveridine; AUC_0-∞_, the area under the plasma concentration–time curve from 0 to ∞; BW, body weigh; CID, collision-induced dissociation; CDL, curved desorption line; Cl_B_, total body clearance; *C*_max_, maximum drug concentration; D1, 3′-desmethyl-DVD; D2, monoglucuronide of 3′-desmethyl-DVD; DVD/D0, diaveridine; HPLC, high-performance liquid chromatography; LC/MS-IT-TOF, liquid chromatography combined with hybrid ion trap/time-of-flight mass spectrometry; LC-LTQ-Orbitrap, high-performance liquid chromatography/linear ion trapped orbitrap; LSC, liquid scintillation counter; MRT, mean residence time; SPE, solid phase extraction; *t*_1/2_, elimination half-life; *T*_max_, time to *C*_max_ from time zero; *v.*ARC, online isotope detector system; *V*_d_, apparent volume of distribution; VICH, International Cooperation on Harmonization of Technical Requirements for the Registration of Veterinary Medicinal Products [↑](#footnote-ref-2)
